# Targeting claudin-4 enhances CDDP-chemosensitivity in gastric cancer

**DOI:** 10.18632/oncotarget.26758

**Published:** 2019-03-15

**Authors:** Yukiko Nishiguchi, Rina Fujiwara-Tani, Takamitsu Sasaki, Yi Luo, Hitoshi Ohmori, Shingo Kishi, Shiori Mori, Kei Goto, Wataru Yasui, Masayuki Sho, Hiroki Kuniyasu

**Affiliations:** ^1^ Department of Molecular Pathology, Nara Medical University, Kashihara, Nara 634-8521, Japan; ^2^ Department of Surgery, Nara Medical University, Kashihara, Nara 634-8522, Japan; ^3^ Jiangsu Province Key Laboratory of Neuroregeneration, Nantong University, Nantong, Jiangsu 226001, China; ^4^ Department of Molecular Pathology, Hiroshima University Graduate School, Hiroshima 734-8551, Japan

**Keywords:** claudin, tight junction

## Abstract

Claudins are major tight-junction proteins that mediate cellular polarity and differentiation. The present study investigated whether the 4D3 antibody to the human CLDN4 extracellular domain (that we previously established) is capable of modulating chemotherapeutic sensitivity in gastric cancer (GC). The results of the present study showed that CLDN4 was overexpressed in 137 of the 192 analyzed GC cases, and that CLDN4 expression was retained in tumors of a lower histological grade (more differentiated), and/or those that were caudal-type homeobox protein 2 (CDX2)-positive, but was reduced in more highly undifferentiated, and CDX2-negative GC cases. The study also compared the synergic effects of combining 4D3 with CDDP treatment and knocking down *CLDN4* expression in MKN74 and TMK-1 human GC cells. Co-treatment with 4D3 increased anti-tumor effects of CDDP, whereas *CLDN4* knockdown did not. In the TMK-1 cells, non-tight junction CLDN4 associated with integrin β1, increasing stem cell-associated proteins via FAK-c-SRC signals. The anti-tumoral effect of CDDP and 4D3 was examined in a nude mouse subcutaneous tumor model. In the two GC cell lines, concurrent treatment with 4D3 and CDDP synergistically inhibited cell proliferation and increased tumor necrosis and apoptosis to a greater degree than CDDP treatment alone. These findings suggest that 4D3 might increase chemotherapeutic sensitivity by evoking structural disintegration of tight-junction CLDN4 expressed in gastric cancer.

## INTRODUCTION

Gastric cancer is currently the third most common cause of cancer-related death in Japan [1]. While the overall 5-year survival rate for the disease has increased to 73.1% due to improved methods for early diagnosis, and increased patient access to endoscopic treatment [2], advanced gastric cancer cases (stages III or IV) are still characterized by a poor prognosis, (5-year survival rate, 47.2% and 7.3%, respectively) [2]. A recent study emphasized the importance of multidisciplinary treatment in gastric cancer, particularly including key therapeutic agents, cis-diamminedichloroplatinum (CDDP), 5-fluorouracil (5-FU) and taxans [3]. In Japanese gastric cancer treatment guideline, CDDP plus S-1 (tegafur/gimeracil/oteracil) therapy is recommended as the standard 1st line chemotherapy for gastric cancer [3]. As the first treatment at the present time, CDDP plus S-1 therapy is considered to contribute most to the extension of survival time. Capecitabine plus CDDP and S-1 plus docetaxel are the second recommended protocols. We have reported that anti-claudin-4 (CLDN4) antibody enhances anti-tumoral effect of 5-FU by reducing barrier function of the tight junction in colorectal cancer [4]. In the present study, we are prompted to evaluate sensitizing effect of CDDP in gastric cancer.

Tight junctions are cellular junctions that are known to mediate cellular polarity and differentiation [5, 6], and to critically control paracellular substance transportation [7]. The claudin family of tight-junction proteins comprises 27 highly homologous members, which are widely expressed, and that have been shown to play essential and integral roles in tight junction formation [5, 7]. For example, CLDN4 is an epithelial claudin that mediates the formation of tight junctions in the mucosal epithelium of the digestive and urinary tracts [5, 7, 8]. In fact, CLDN4 overexpression has been reported in both normal mucosal epithelia, and a wide variety of human malignancies [9–12]. Furthermore, CLDN4 expression is associated with disease progression in, and thus has been suggested as a promising therapeutic target for ovarian, bladder, pancreatic, and colorectal cancer [9–13].

Unfortunately, creating antibodies to specifically target the extracellular domain of any one particular claudin protein is difficult because this region is highly homologous among the claudin family members. Therefore, we previously established an antibody to target the human CLDN4 extracellular domain, by conditionally expressing human *CLDN4* by using a published DNA immunization technique [12–14]. Importantly, we showed that the generated anti-CLDN4 antibody (clone 4D3) was capable of enhancing anti-cancer effects induced by CDDP treatment in bladder cancer [13]. Thus, the present study investigated whether the same antibody also enhances CDDP-associated anti-cancer effects in gastric cancer.

## RESULTS

### CLDN4 expression in gastric cancer

**Figure 1:**
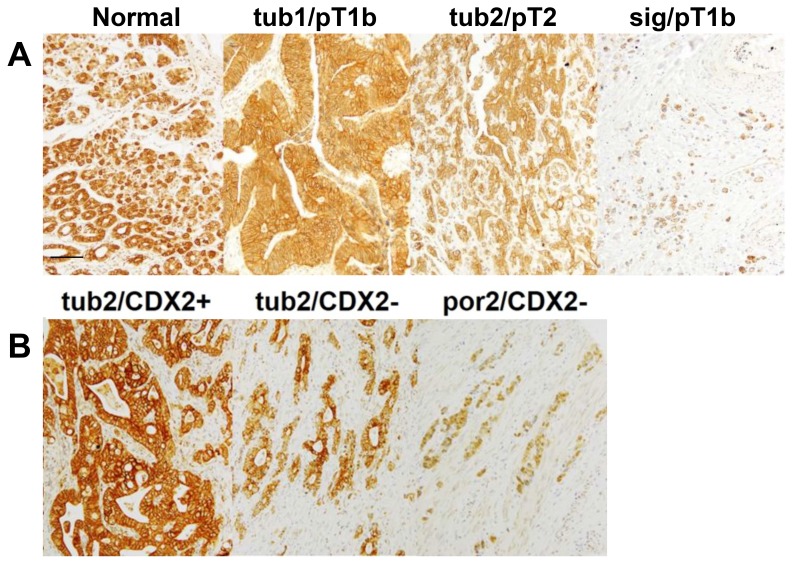
CLDN4 expression in gastric adenocarcinomas (**A**) A conducted immunohistochemical evaluation identified CLDN4 expression at the cytoplasmic membrane of the normal gastric mucosa, and in both the cytoplasmic membrane and cytoplasm of three representative gastric cancer cases. Notably, undifferentiated cancer cases exhibited lower CLDN4 expression levels than differentiated cases. (**B**) A comparison of CLDN4 expression levels and patient CDX2 statuses showed that CLDN4 expression was lower in CDX2(–) than CDX2(+) cases. Bar, 100 μm. Tub1, well-differentiated tubular adenocarcinoma; tub2, moderately differentiated tubular adenocarcinoma; sig, signet-ring cell carcinoma; por2, poorly differentiated adenocarcinoma; pT1b, invasion into the submucosal layer; pT2, invasion into the muscle propria layer.

An immunohistochemical examination of CLDN4 expression in the 192 analyzed gastric cancer cases showed that, while CLDN4 immunoreactivity was restricted to the cytoplasmic membrane in the normal gastric epithelium, it was observed both at the cytoplasmic membrane, and in the cytoplasm of gastric cancer cells (Figure 1A). Notably, CLDN4 expression levels were progressively reduced in tumor tissues of higher histological grades (i.e. those that exhibited lower levels of differentiation) (Figure 1A, Table 1). Furthermore, CLDN4 expression was inversely correlated with tumor invasion (pT), nodal metastasis (pN) distant metastasis (pM), and pathological staging (pStage) (Table 1).

**Table 1:** Relationship between CLDN4 expression and clinicopathological parameters in 192 gastric cancer cases

Parameters^1^	*n*	Claudin-4 expression index^2^	*P*^3^
Age			
< 60yo	61	194 ± 14	
≥ 60yo	131	189 ± 17	NS^4^
Sex			
Male	118	191 ± 15	
Female	74	193 ± 17	NS
Histological classification			
pap	29	170 ± 15	
tub1	24	216 ± 15	
tub2	59	198 ± 11	
por1	16	186 ± 20	
por2	49	130 ± 11	
sig/muc	15	121 ± 19	
Differentiated type (pap, tub1, tub2)	112	195 ± 15	
Undifferentiated type (por1, por2, sig, muc)	80	139 ± 17	< 0.0001
pT			
1–2	138	180 ± 7	
3–4	54	148 ± 11	< 0.05
pN			
0	168	169 ± 7	
1–3	24	184 ± 18	NS
M			
0	190	172 ± 6	
1	2	90 ± 50	NS
pStage			
I	127	178 ± 7	
II–IV	65	159 ± 11	NS

^1^Histological and clinicopathological classification were according to Japanese Gastric Cancer Classification [40]: pap, papillary adenocarcinoma; tub1, well-differentiated tubular adenocarcinoma; tub2, moderately differentiated tubular adenocarcinoma; por1, solid type poorly differentiated adenocarcinoma; por2, non-solid type poorly differentiated adenocarcinoma. sig, signet-ring cell carcinoma; muc, mucinous adenocarcinoma; pT1, tumor confined to the mucosa or the submucosa; pT2, tumor invades the muscularis propria; pT3, tumor invades the subserosa; pT4, tumor invasion is contiguous to or exposed beyond the serosa or tumor invades adjacent structures; pN0, no regional lymph node metastasis; pN1, metastasis in 1–2 regional lymph nodes; pN2–3, metastasis in 3 or more regional lymph nodes; M0, no distant metastasis; M1, distant metastasis; pStage I, pT1 and pN0 or pN1, or pT2 and pN0, pStage II–IV, all cases other than pStage I.

^2^Claudin-4 expression index was calculated as the staining strength score multiplied by the staining area.

^3^*P* value was calculated by ordinary ANOVA test. NS, not significant.

### Relation of CLDN4 expression with clinicopathological parameters in differentiated and undifferentiated gastric cancer types

Given that CLDN4 expression varied with tumor histological grading, its association with other clinicopatholgical parameters was assessed by classifying the analyzed cases as either ‘differentiated’ or ‘undifferentiated’ (Table 2). In the differentiated cases (i.e. pap, tub1, and tub2 cases, Figure 1) there was no correlation between CLDN4 expression and disease progression (i.e. pT, pN, and pStage). In contrast, among the undifferentiated cases (i.e. por1, por2, sig, and muc cases, Figure 1) CLDN4 expression was inversely correlated with pT.

**Table 2:** Relationship between CLDN4 expression and clinicopathological parameters in differentiated and undifferentiated types of gastric cancer

	Differentiated cases^1^			Undifferentiated cases^2^		
	*n*	Expression index^3^	*P*^4^	*n*	Expression index^3^	*P*^4^
Parameters^5^						
pT						
1–2	85	195 ± 9		45	157 ± 12	
3–4	27	190 ± 12	NS	25	107 ± 17	< 0.05
pN						
0	96	194 ± 8		63	135 ± 10	
1–3	16	198 ± 21	NS	7	174 ± 37	NS
pStage						
I	77	196 ± 10		43	149 ± 12	
II–IV	35	190 ± 13	NS	27	123 ± 17	NS

^1^Differentiated gastric cancer contains papillary adenocarcinoma, well-differentiated tubular and moderately differentiated tubular adenocarcinoma.

^2^Undifferentiated gastric cancer contains poorly differentiated adenocarcinoma, signet-ring cell carcinoma and mucinous adenocarcinoma.

^3^Claudin-4 (CLDN4) expression index was calculated as the staining strength score multiplied by the staining area.

^4^*P* value was calculated by ordinary ANOVA test. NS, not significant.

^5^pT1–2, tumor confined within the submucosa or tumor invades the muscularis propria; pT3–4, tumor invades the subserosa or tumor invasion is contiguous to or exposed beyond the serosa or tumor invades adjacent structures; pN0, no regional lymph node metastasis; pN1–3, metastasis in 1 or more regional lymph nodes; pStage I, pT1 and pN0 or pN1, or pT2 and pN0, pStage II–IV, all cases other than pStage I [34].

### Relation of CLDN4 expression with clinicopathological parameters in caudal-type homeobox protein 2 (CDX2)-positive and -negative gastric cancer

Epithelial claudin expression is modulated by Helicobacter pylori infection [15]; therefore, the analyzed cancer cases were divided into CDX2(+) and CDX2(–) cases (Tables 3 and 4). The CDX(+) cases were found to be predominantly differentiated, whereas conversely, CDX(–) cases were predominantly undifferentiated (Table 3). Overall, CLDN4 expression was higher in CDX(+) (index value, 181.0 ± 17.8) than CDX(–) cases (157.2 ± 19.7) (Table 4). For example, as shown in Figure 1B, a CDX(+) tub2 case exhibited higher CLDN4 expression levels than a CDX(–) tub2 case. Notably, while no correlation between CLDN4 expression and disease progression (pT, pN, and pStage) was identified among the CDX(+) cases, CLDN4 expression was inversely correlated with pT and pStage in CDX(–) cases.

**Table 3:** Relation of CDX2 expression with histological classification of gastric cancer cases

		*n*	Histological classification^1^					
			pap	tub1	tub2	por1	por2	sig/muc
CDX2^2^	(+)	116	29	51	24	3	6	3
	(–)	76	0	0	8	13	43	12

^1^Histological classification is according to Japanese Gastric Cancer Classification [34]: pap, papillary adenocarcinoma; tub1, well-differentiated tubular adenocarcinoma; tub2, moderately differentiated tubular adenocarcinoma; por1, solid type poorly differentiated adenocarcinoma; por2, non-solid type poorly differentiated adenocarcinoma. sig, signet-ring cell carcinoma; muc, mucinous adenocarcinoma.

^2^CDX2 was evaluated as positive by immunoreactivity in 20% or more nuclei of 1000 observed nuclei. Distribution of histological differentiation was significantly different between CDX2(–) and CDX2(+) cases (*P* < 0.0001).

**Table 4:** Relationship between CLDN4 expression and clinicopathological parameters in CDX2-positive and -negative gastric cancer

	CDX2(+)^1^			CDX2(–)^1^		
	*n*	Expression index^2^	*P*^3^	*n*	Expression index^2^	*P*^3^
Parameters^4^						
pT						
1–2	82	182 ± 10		56	178 ± 11	
3–4	34	179 ± 14	NS	20	99 ± 15	< 0.001
pN						
0	99	177 ± 9		69	159 ± 10	
1–3	17	202 ± 20	NS	7	141 ± 38	NS
pStage						
I	75	183 ± 10		52	172 ± 11	
II–IV	41	177 ± 13	NS	24	126 ± 17	< 0.05

^1^CDX2 was evaluated as positive by immunoreactivity in 20% or more nuclei of 1000 observed nuclei.

^2^Claudin-4 expression index was calculated as the staining strength score multiplied by the staining area.

^3^*P* value was calculated by ordinary ANOVA test. NS, not significant.

^4^pT1–2, tumor confined within the submucosa or tumor invades the muscularis propria; pT3–4, tumor invades the subserosa or tumor invasion is contiguous to or exposed beyond the serosa or tumor invades adjacent structures; pN0, no regional lymph node metastasis; pN1-3, metastasis in 1 or more regional lymph nodes; pStage I, pT1 and pN0 or pN1, or pT2 and pN0, pStage II–IV, all cases other than pStage I [34].

### Effect of the 4D3 antibody on human gastric cancer cells *in vitro*

**Figure 2:**
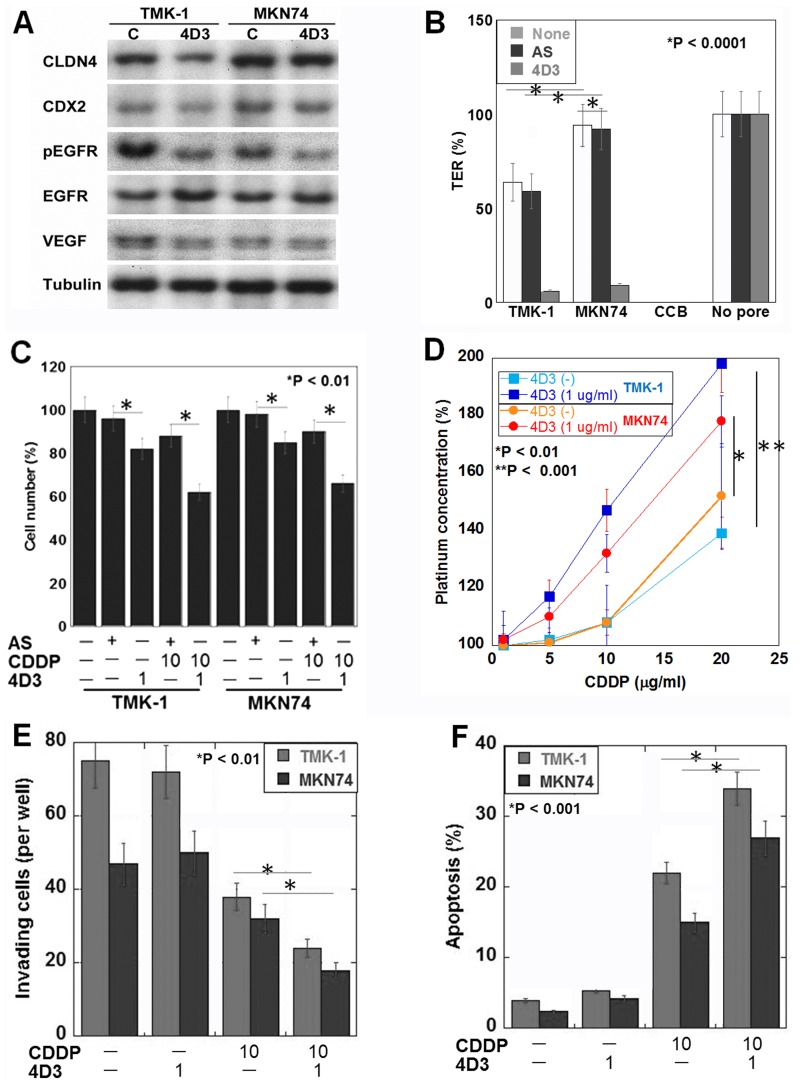
Effects induced by the 4D3 antibody in gastric cancer cells *in vitro* (**A**) CLDN4, CDX2, EGFR, phosphorylated EGFR, and VEGF expression levels were examined by Western blotting. Tubulin was used as a loading control. (**B**) The transepithelial electrical resistance (TER) of 4D3-treated, and CLDN4 knockdown cells was measured. Cytochalasin B (CCB) was used to dissociate cells (negative control). (**C**) The combined effects of CDDP treatment and either 4D3 treatment or CLDN4 knockdown were examined. (**D**) The intracellular platinum concentration was measured in cells with or without 4D3 treatment. (**E**, **F**) (Transwell) invasion and apoptotic behaviors, respectively, were examined in cells treated with CDDP and/or 4D3. The S.D. was calculated from three independent trials.

Next, we examined the effect of 4D3 on gastric cancer cells using poorly differentiated adenocarcinoma-derived TMK-1 cells, and well-differentiated adenocarcinoma-derived MKN74 cells (Figure 2). The results of this analysis showed that the MKN74 cells expressed CLDN4 and CDX2 at higher levels than the TMK-1 cells (Figure 2A). By 4D3 treatment *CLDN4* expression decreased CLDN4 production levels and the levels of phosphorylated EGFR and VEGF in both cell types. A TER examination of tight junction integrity showed firstly that the MKN74 cells exhibited a higher TER than the TMK-1 cells (Figure 2B), and secondly that in both cell types, 4D3 treatment, but not CLDN4 knockdown, decreased the TER. Likewise, cell proliferation was inhibited by 4D3 treatment, but not by CLDN4 knockdown in both cell lines (Figure 2C). Importantly, co-treatment with CDDP and 4D3 induced a more pronounced inhibitory effect than that observed during co-treatment with CDDP and CLDN4 knockdown. When the gastric cancer cells were treated with a combination of 4D3 and CDDP, intracellular platinum concentrations were increased to 1.5- and 1.2-times the levels seen in CD74 and TMK-1 cells, respectively, treated with CDDP alone (Figure 2D). Co-treatment with CDDP and 4D3 had synergic effect on the inhibition of invasion and induction of apoptosis in the two cell lines (Figure 2E and 2F).

### Effect of 4D3 on non-tight junction CLDN4 in TMK-1 cells

**Figure 3:**
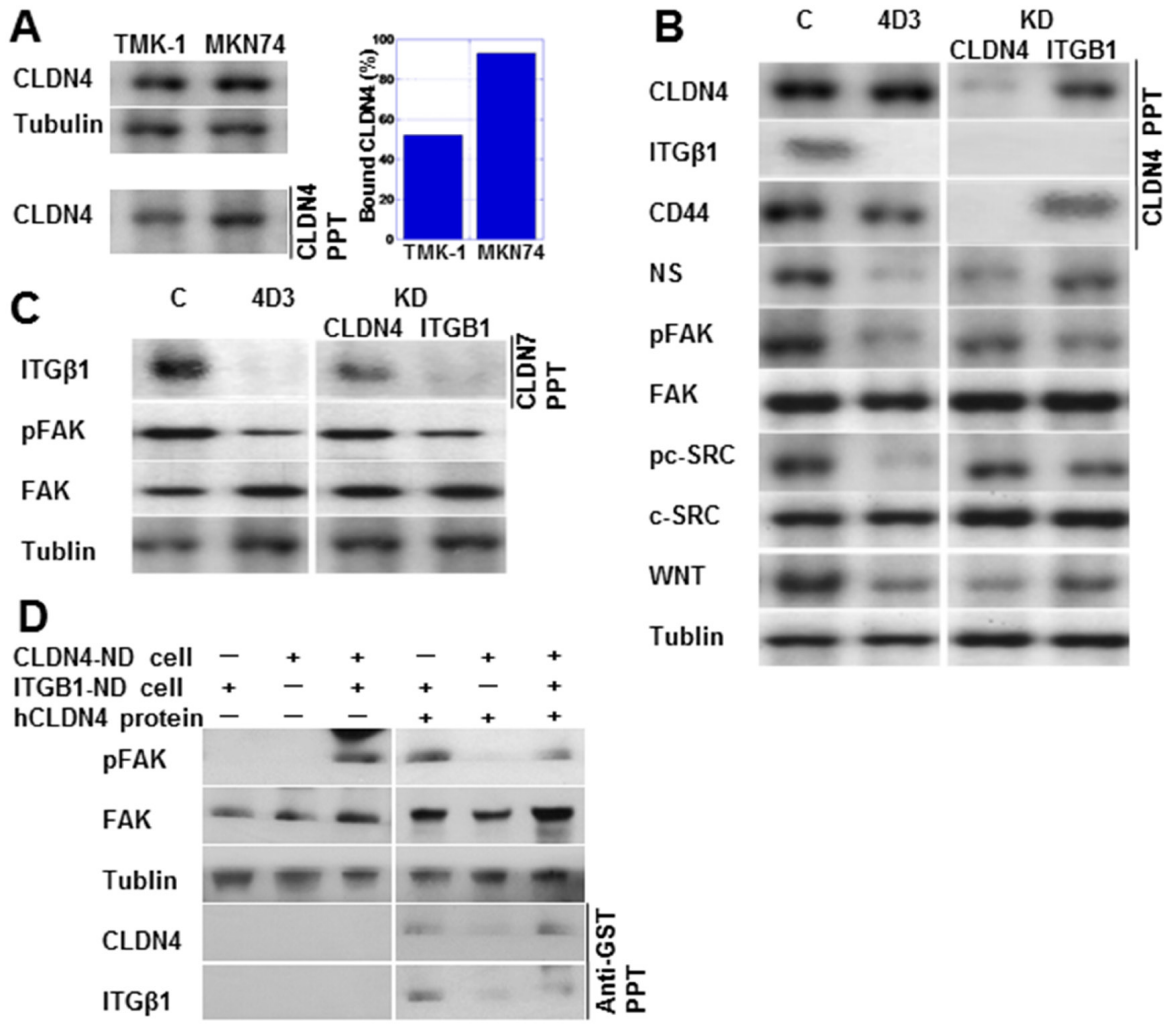
4D3 treatment inhibited the interaction between CLDN4 and integrin β1 (ITGβ1) (**A**) CLDN4-CLDN4 interaction was investigated using immunoprecipitation. The amount of bound CLDN4 was calculated as the quotient of CLDN4 levels in precipitants divided by their levels in whole lysates (standardized by tubulin). (**B**) Physical interaction between CLDN4 and ITGβ1 or CD44 was examined using immunoprecipitation. Expression of integrin signal proteins and stemness-associated proteins was also examined in TMK-1 cells treated with 4D3 or by CLDN4 or ITGβ1 knockdown. (**C**) Physical interaction between CLDN7 and ITGβ1 was examined by immunoprecipitation in TMK-1 cells treated with 4D3 or by CLDN4 or ITGβ1 knockdown. (**D**) Integrin signal was rescued in CLDN4(–)/ITGβ1(+) TMK-1 cells by CLDN4 not associated with cells. Tubulin was used as a loading control. NS, nucleostemin; FAK, focal adhesion kinase; pFAK, phosphorylated FAK; pc-SRC, phosphorylated c-SRC; WNT, wingless-related integration site.

As shown in Figure 3A, immunoprecipitation analysis showed lower levels of the bound form of CLDN4 in TMK-1 compared to MKN74 cells. Notably, in the TMK-1 cells, CLDN4 was co-immunoprecipitated with integrin β1 (ITGβ1) as well as CLDN4, with these effects abrogated by knockdown of CLDN4 or ITGβ1, respectively (Figure 3B). In contrast, CD44 was co-immunoprecipitated with CLDN4, which was not affected by ITGβ1 knockdown. With 4D3 treatment, expression levels of ITGβ1 and its downstream proteins, pFAK and pc-SRC, were decreased. Expression levels of stem cell-associated proteins CD44, NS, and WNT were also decreased by 4D3 treatment. In contrast, knockdown of CLDN4 partially inhibited ITGβ1 signal activation in comparison to 4D3 treatment. CLDN7 is reported to bind to ITGβ1 [16]. In Figure 3C, ITGβ1 is seen co-precipitated with CLDN7, with the ITGβ1 signal being retained in CLDN4-knockdown TMK-1 cells. CLDN4 was not physically associated with CLDN7.

To confirm that non-tight junction CLDN4 is associated with ITGβ1, CLDN4-knockdown TNK-1 cells (CLDN4-KD cells) and ITGβ1-knockdown TMK-1 cells (ITGβ1-KD cells) were cocultured (Figure 3D). The integrin signal (pFAK) was activated in the cocultured cells, but not in either CLDN4-KD or ITGβ1-KD cells alone. Addition of GST-tagged recombinant CLDN4 protein activated pFAK in CLDN4-KD cells, whereas CLDN4 protein decreased pFAK levels in the cocultured cells. GST-tagged CLDN4 was co-precipitated with ITGβ1.

### *In vivo* effect of 4D3 on human gastric cancer cells

**Figure 4:**
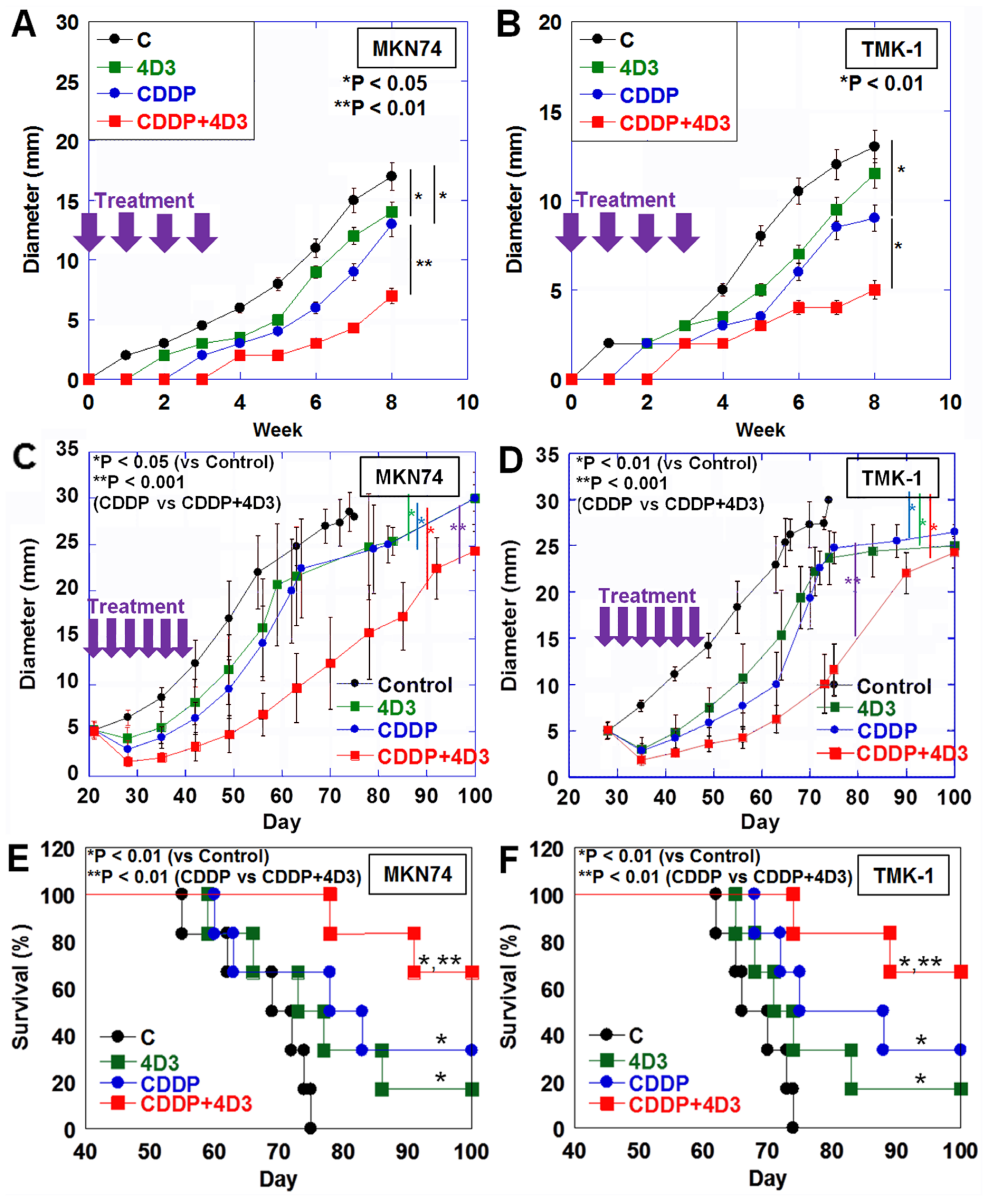
Effect of concurrent treatment with CDDP and 4D3 on growth of gastric cancer cells in nude mice In nude mice, MKN74 and TMK-1 tumors were treated with CDDP and/or 4D3. (**A**, **B**) Tumors were treated with CDDP (3 mg/kg BW) and/or 4D3 (1 mg/kg BW) on Day 1, 8, 15, and 22. (**C**, **D**) After MKN74 and TMK-1 tumors were grown to a diameter of 5 mm, they were treated with CDDP (3 mg/kg BW) and/or 4D3 (1 mg/kg BW) 3 times a week for 2 weeks. (**E**, **F**) Survival of mice with MKN74 or TMK-1 tumors (as seen in Figure 4C and 4D) were calculated using the Kaplan–Meier method. Bar, S.D.

We next examined the effect of 4D3 on human gastric cancer cell tumor growth in a nude mouse subcutaneous tumor model (Figure 4).

In the first experiment, tumor-burden mice were treated with CDDP and/or 4D3 on the same day as inoculation (Figure 4A, 4B). Treatment with 4D3 alone significantly inhibited growth of MKN74 tumors, but not TMK-1 tumors, in comparison with control mice. In contrast, treatment with CDDP alone inhibited tumor growth significantly in both MKN74 and TMK-1 tumors. Concurrent treatment with CDDP and 4D3 showed a more pronounced inhibitory effect against both MKN74 and TMK-1 tumors than treatment with CDDP alone. Concurrent treatment with CDDP and 4D3 showed a synergic inhibitory effect against both cells (synergic indices were 4.2 and 3.9 in MKN74 and TMK-1, respectively). The effect of concurrent treatment with CDDP and 4D3 on tumors was examined after 8 weeks (Table 5). Necrotic area and apoptotic changes (ss-DNA and cleaved caspase-3) were greater in tumors treated with CDDP and 4D3 than in tumors treated with CDDP alone. In contrast, cell proliferation (Ki-67) was decreased in tumors treated with CDDP and 4D3, compared to those treated with CDDP alone.

**Table 5:** Effect of 4D3 on inhibitory effect of CDDP in tumors of MKN74 and TMK-1 cells

	TMK-1				MKN74			
CDDP	−	+	+	*P*^4^	−	+	+	*P*^4^
4D3	−	−	+		−	−	+	
Necrotic area (%)^1^	2 ± 0.1	27 ± 3	48 ± 5	< 0.001	14 ± 2	52 ± 7	68 ± 13	< 0.001
Ki-67 (%)^2^	88 ± 4	78 ± 3	75 ± 3	< 0.05	92 ± 5	81 ± 4	76 ± 4	< 0.05
ss-DNA (%)^2^	12 ± 1	23 ± 2	34 ± 3	< 0.05	8 ± 1	22 ± 1	34 ± 2	< 0.05
Cleaved caspase-3 (/HPF)^3^	1 ± 0.1	4 ± 0.1	6 ± 0.2	< 0.001	2 ± 0.1	4 ± 0.1	10 ± 0.2	< 0.001

^1^Necrotic area was evaluated in computer-captured images of the maximum cut surface of the tumors.

^2^Positivity was evaluated as positive by immunoreactivity in 20% or more nuclei of 1000 observed nuclei.

^3^Positive cells were counted by observation of 20 high power fields (HPFs).

^4^*P* value was calculated by ordinary ANOVA test.

In the second set of experiments conducted using the mouse model, tumors grown to a diameter of 5 mm were treated with CDDP and/or 4D3 (Figure 4C, 4D). Treatment with 4D3 or CDDP alone inhibited tumor growth significantly in comparison with control mice in both MKN74 and TMK-1 tumors. Concurrent treatment with CDDP and 4D3 showed a more pronounced inhibitory effect in both MKN74 and TMK-1 tumors than did treatment with CDDP alone. Moreover, concurrent treatment with CDDP and 4D3 showed a synergic effect against both cell types (synergic indices were 5.1 and 4.9 in MKN74 and TMK-1, respectively). The survival analysis of the mice is shown in Figure 4E and 4F. For both cell lines, mice treated with 4D3 or CDDP alone survived for longer than control mice. Concurrent treatment with CDDP and 4D3 improved their survival even further.

## DISCUSSION

We investigated the effectiveness of anti-CLDN4 extracellular domain antibody 4D3 as a new molecular-targeted drug against gastric cancer using pathological specimens, as well as *in vitro* and *in vivo* experiments.

Under normal conditions, gastric mucosal epithelium is SOX2-positive and CDX2-negative, but is changed to SOX2-negative and CDX2-positive by *H. pylori* infection [17–19]. CDX2-positive gastric cancer is more prevalent in differentiated type gastric cancers. In these differentiated/CDX2-positive gastric cancer cases, CLDN4 is at high levels than undifferentiated/CDX2-negative cases. CDX2 expression is related to CLDN4 expression. In differentiated/CDX2-positive gastric cancer, CLDN4 expression is not associated with disease progression, but is inversely associated with disease progression in undifferentiated/CDX2-negative cases. These data suggest that the role of CLDN4 might be different between differentiated/CDX2-positive and undifferentiated/CDX2-negative gastric cancers.

*H. pylori* have a more pronounced effect on differentiated than on undifferentiated gastric cancers [20]. It is thought that overexpression of CLDN4 caused by *H. pylori* might result in the formation of tight junctions, which might provide an isolated tumor microenvironment mediated through the barrier function of the tight junction. In this microenvironment, an accumulation of growth factors and inhibition of permeation by anti-cancer agents has previously been observed [13].

CLDN4, an epithelial phenotype marker [5, 7, 8], is decreased during epithelial–mesenchymal transition (EMT) [21]. Decreases in CLDN4 expression in undifferentiated/CDX2-negative gastric cancer might be explained as resulting from induction of the EMT phenotype. Indeed, it is reported that EMT is enhanced in undifferentiated gastric cancers [21].

We have reported that TNFα decreases CLDN4 expression in colorectal cancer [4]. Although inflammatory cytokines enhance remodeling of tight junction, their effects on claudin expression depend on types of cytokines and cells [22, 23]. In gastric cancer, overexpression and the polymorphism of interleukin (IL)-1β are well known [24, 25]. IL-1α, IL-1β, and IL-18 downregulates CLDN4 protein by disrupting tight junction via activation of IL-1 receptor-Rho pathway in gastric epithelial cells [26]. CLDN18 knockout mice show prompted onset of atrophic gastritis by disrupted proton barrier with IL-1β upregulation [27]. Thus *H. pylori* infection impairs tight junction and activation of inflammatory cytokines. In contrast, our data showed that CLDN4 was overexpressed in *H. pylori*-associated differentiated type gastric cancer. Long exposure to *H. pylori* lipopolysaccharide activates toll-like receptor 2 to upregulate expression of CLDN4, 6, 7 and 9 [28]. It is suggested that difference in duration of *H. pylori* infection might cause difference in effect: atrophic gastritis and initiation of gastric carcinogenesis with CLDN4 downregulation might precede differentiated type gastric cancer with CLDN4 overexpression.

Importantly, in our study, CLDN4 not assembled into tight junctions was associated with ITGβ1, generating an intracellular signal associated with increased stemness. TMK-1 cells expressed CLDN4 at lower levels than did MKN74 cells. Furthermore, TER expression was decreased to a greater degree than CLDN4 expression. Moreover, CLDN4 levels were lower in TMK-1 cells than in MKN74 cells. These findings suggest that CLDN4 outside of tight junction assembly was present in greater quantities in TMK-1 cells than in MKN74 cells. Physical interaction between CLDN4-KD TMK-1 cells and ITGβ1-KD TMK-1 cells activated the integrin signal; however, the signal was decreased following treatment with recombinant CLDN4. Therefore, the non-tight junction CLDN4 was bound to ITGβ1, increasing stem cell markers such as nucleostemin or WNT. Our data also showed that CD44 physically associated with CLDN4, an association that has been previously suggested [29]. Thus, CLDN4 might play a role in integrin signaling, or in CD44 associated with stemness in undifferentiated/CDX2-negative gastric cancer. CLDN7, an epithelial claudin [8], is associated physically with ITGβ1 [16]. In TMK-1 cells, CLDN7-ITGβ1 association was detected; however, CLDN4-ITGβ1 provided a greater activated integrin signal.

In this study, we investigated the anti-tumoral effect of anti-human CLDN4 extracellular domain antibody in comparison to the effect of CLDN4 knockdown. The anti-tumoral effect was more pronounced, following antibody treatment, than was CLDN4 knockdown, either with or without CDDP. It is suggested that removal of CLDN4 by knockdown induced other epithelial claudins to be enhanced in order to maintain tight junctions. In contrast, anti-CLDN4 antibody impairs tight junction formation and the CLDN4-ITGβ1 relationship without supplementation by other claudins.

In bladder cancer, expression of CLDN4 is associated with chemotherapeutic resistance, and anti-CLDN4 antibody increases chemotherapeutic sensitivity [13]. In our *in vivo* examination, anti-tumoral effects were more pronounced in groups treated with CDDP and anti-CLDN4 antibody than in groups treated with CDDP alone. We confirmed that wider tumor necrosis, improved suppression of tumor growth, and improved apoptosis occurred in groups treated with CDDP and the anti-CLDN4 antibody than in other groups. These anti-tumoral findings were more pronounced MKN74 cell tumors, which showed higher expression of CLDN4 than did undifferentiated gastric cancer TMK-1 cells. Non-tight junction CLDN4 is plentiful in undifferentiated type gastric cancer to activated integrin β1, which increases stemness to provide metastability anti-apoptotic survival. In contrast, tight junction CLDN4 is plentiful in differentiated type gastric cancer to form anti-cancer drug barrier. Levels of non-tight junction CLDN4 might be affect malignant potential of differentiated type gastric cancer. Nevertheless, as the anti-tumoral mechanism of 4D3 is in effect irrespective of whether abrogation of tight junction occurs, or whether the CLDN4-ITGβ1 relationship is inhibited, higher expression of CLDN4 might be responsible for the higher anti-tumoral effect to gastric cancer. High CLDN4 expression in gastric cancer cases suggests that CLDN4 targeting by anti-CLDN4 antibody might be a relevant tool for treatment.

In molecular targeted therapies, it is important that potential side effects on normal tissues be investigated. In this study, we injected anti-CLDN4 antibody into nude mice, so we cannot investigate its effect on normal mice. CLDN4 expression is important for epithelial tight junction formation throughout the whole body, especially in the intestinal tract [5, 7, 8]. As antibodies are administered inject by intravenous injection, some research has suggested that it is difficult for them to reach tight junctions on the surface of intestinal mucosa [30]. We investigated possible side effects by injecting an antibody that recognizes both mouse and human antigen into mice tumor models [31]. The anti-claudin antibody is able to reach the intestinal tract and associated tumor tissue [31]. It also reached the liver and kidney, but both organ's main tight junction is claudin-1, so we did not detect severe side effects [31–33]. In the human body, it is suggested that antibodies are able to reach tumors, the liver, and kidney, and can enhance CDDP anti-cancer effects while having few side effects. Anti-CLDN4 antibody has potential application as a new type of molecular targeted drug capable of enhancing the anti-cancer properties of existing drugs, and in the development of molecular targeted therapies against gastric cancer.

## MATERIALS AND METHODS

### Patients

A total of 192 cases of gastric cancer, which were surgically resected in Miyoshi Central Hospital and histopathologically diagnosed by the Department of Molecular Pathology, Nara Medical University during 2001–2015, were analyzed. As written informed consent was not obtained from patients for their participation in the present study, all identifying information was removed from patient samples prior to their analysis, to ensure strict privacy protection (unlinkable anonymization). All procedures were performed in accordance with the Ethical Guidelines for Human Genome/Gene Research enacted by the Japanese Government, and with the approval of the Ethics Committee of Nara Medical University (Approval Number, 937).

### Cells and reagents

A human gastric cancer cell line, TMK-1, was previously established from a fundic gland-type gastric cancer case [34]. The well-differentiated gastric cancer-derived cell line MKN74 was obtained from the Japanese Collection of Research Bioresources (JCRB; Osaka, Japan). Cells were cultured in Dulbecco's modified Eagle's medium (DMEM; Sigma Chemical Co., St. Louis, MO, USA), that was supplemented with 10% fetal bovine serum (FBS; Sigma). CDDP was purchased from Sigma. All other utilized reagents were of research grade.

### Anti-human CLDN-4 extracellular domain monoclonal antibody

We previously established an anti-human CLDN4 extracellular domain monoclonal antibody, clone 4D3, that specifically recognizes the human CLDN4 extracellular protein domain [13].

### Cell growth and apoptosis

Cell growth was assessed via a tetrazolium (MTT) dye assay, as previously described [35]. Apoptosis was assessed via the examination of 1000 cells, which were stained with Hoechst 33342 dye (Life Technologies, Carlsbad, CA, USA), and viewed using a fluorescent microscope.

### Chamber invasion assay

A modified Boyden chamber assay was performed to examine the *in vitro* invasion of colon cancer cells [36]. Following incubation at 37°C for 24 h, the filters were carefully removed from the inserts, stained with hematoxylin for 10 min, and mounted on microscopic slides. The number of stained cells in each insert was counted at 100× magnification. Invasion activity was quantified by calculating the average number of cells per insert well. These experiments were performed in triplicate.

### Integrin β1-CLDN4 binding assay

*CLDN4* or *ITGB1* expression was knocked down in TMK-1 cells using Stealth Select short interfering RNA (siRNA) constructs (Invitrogen, Carlsbad, CA). The TMK-1 cells were transfected with *CLDN4, ITGB1,* and/or Negative (control; Invitrogen) siRNAs (20 nM) using Lipofectamine 2000 (Invitrogen) according to the manufacturer's instructions. Induced effects on gene expression were confirmed via real-time RT-PCR. Cultures of *CLDN4*- (*CLDN4*-KD; 2 × 10^5^), *ITGB1*-knockdown (*ITGB1*-KD; 2 × 10^5^), and both *CLDN4*-KD and *ITGB1*-KD (each 1 × 10^5^) TMK-1 cells were maintained with or without recombinant human CLDN4 protein (50 μg/mL; with GST tag, ab114389, Abcam) for 24 h, before being harvested to extract whole cell lysates.

### Animals

Four-weeks-old BALB/c Slc-nu/nu mice were purchased from SLC Japan, Inc. (Shizuoka, Japan), and maintained in accordance with both the institutional guidelines approved by the Committee for Animal Experimentation of Nara Medical University, and the current regulations and standards of the Ministry of Health, Labor, and Welfare (Approval number 11569 and 11596).

### Animal tumor models

To establish a subcutaneous tumor model, cancer cells (1 × 10^7^) were inoculated into the scapular subcutaneous tissues of nude mice, and the resultant tumors were observed for 4 weeks. CDDP (3 mg/kg body weight, diluted with saline) and/or 4D3 (1 mg/kg body weight, diluted with saline) were then simultaneously injected into the subcutaneous tissue. The synergic effect of this treatment was assessed using the following formula [37]:

Synergy index = {(AUC_Control_ – AUC_CDDP+4D3_)/(AUC_Control_ – AUC_CDDP_)} + {(AUC_Control_ – AUC_CDDP+4D3_)/(AUC_Control_ – AUC_4D3_)}.

Where AUC is the area under the tumor growth curve. An index value >2 was considered to indicate a synergic effect.

### Immunohistochemistry

Consecutive 4-μm sections were immunohistochemically stained using the immunoperoxidase technique described previously [38], with primary anti-CLDN4 (4D3), and appropriate secondary antibodies (Medical and Biological Laboratories {MBL}, Nagoya, Japan) (all 0.2 μg/mL). The tissue sections were then color-developed with diamine benzidine hydrochloride (DAKO, Glostrup, Denmark), and counterstained with Meyer's hematoxylin (Sigma). We counted cells that exhibited immunoreactivity at the cytoplasmic membrane, and scored staining strength between 0 to 3, (where a score of 1 was used to describe the expression level in a normal gastric foveolar epithelium). The staining index was then calculated as the staining strength score multiplied by the staining area (%).

CDX2 expression was assessed immunohistochemically using an antibody to CDX2 (Abcam, Cambridge, UK). Positive CDX2 expression was inferred by the presence of immunoreactivity in 20% or more of 1000 observed nuclei.

Similarly, cell proliferation was assessed immunohistochemically by incubating cells with an antibody to Ki-67 (DAKO), and examining 1000 nuclei for positive antibody staining. Apoptosis was immunohistochemically examined, and identified via dual staining with antibodies to single strand DNA (ss-DNA, MBL) and cleaved caspase-3 (cas3, MBL), in 20 high power fields (HPF).

Histological necrosis was evaluated by measuring necrotic regions in computer-captured images using an analysis application in Photoshop (CS5.1, Adobe Systems Inc., San Jose, CA, USA).

### Immunoblot analysis

Whole-cell lysates were prepared as previously described [37]. Lysates (50 μg) were subjected to an immunoblot analysis using SDS-PAGE (12.5%), and electrotransferred onto nitrocellulose filters. The filters were incubated with primary antibodies for the epidermal growth factor receptor (EGFR; Cell Signaling Technology, Beverly, MA, USA), phosphorylated EGFR (pTyr992) (pEGFR; Cell Signaling Technology), vascular endothelial growth factor (VEGF; SC-374628, Santa-Cruz), nucleostemin (NS; SC-166460, Santa-Cruz), focal adhesion kinase (FAK; SC-271126, Santa-Cruz), phosphorylated FAK (pTyr397) (pFAK; P00151, Boster Immunoleader, Pleasanton, CA, USA) c-SRC (25978-1-AP, Proteintech Group, Rosemont, IL, USA) phosphorylated c-SRC (pTyr530) (pc-SRC; SC-166860, Santa-Cruz), and CDX2 (Oncogene Research Products, Cambridge, MA, USA). They were then incubated with peroxidase-conjugated IgG antibodies (MBL), and visualized using an enhanced chemiluminescence Western-blot detection system (Amersham, Aylesbury, UK). An anti-tubulin antibody was used as a loading control (Oncogene Research Products, Cambridge, MA, USA).

### Immunoprecipitation

Immunoprecipitation was performed according to the method described previously [39]. Lysates were pre-cleaned in lysis buffer with protein A/G agarose (Santa Cruz) for 1 h at 4°C and subsequently centrifuged. The supernatants were incubated with a precipitation antibody (either to CLDN4 (4D3), CLDN7 (SC-17670, Santa-Cruz), or GST (ab19256, Abcam)), and protein A/G agarose, for 3 h at 4°C. Precipitates were collected via centrifugation, washed five times with lysis buffer, solubilized with sample buffer (Sigma, 40 μg), and subjected to an immunoblot analysis with antibodies to CLDN4 (4D3), integrin β1 (SC-374429, Santa-Cruz), and/or CD44 (SC-7297, Santa-Cruz).

### Antisense phosphorothioate (S)-oligodeoxynucleotide (ODN) assay

An 18-mer S-ODN antisense sequence (5′-TAG CCC CAT GGA GGC CAT-3′; Genbank NM_001305.4) corresponding to nucleotides 1–18 of the human *CLDN4* gene was synthesized and purified (Sigma-Genosys, Ishikari, Japan). A mixed-sequence 18-mer S-ODN was used as a control. Cells were pretreated with 6 μM antisense or mixed S-ODN for 48 h, before being subjected to additional manipulations.

### Transepithelial electroresistance (TER)

The cellZscope tight junction monitoring system (Fujifilm, Tokyo, Japan) was used to measure the TER of the analyzed cells (1 × 10^5^ cells, seeded onto the provided insert, and allowed to form multiple cell layers). A negative control scenario was generated, in which tight junction formation was impaired via treatment with cytochalasin B (CB; 10 μM, Wako).

### Intracellular platinum

Cells (1 × 10^10^) were digested with proteinase K for 2 h at 45°C, and treated with 65% nitric acid overnight at 80°C. The platinum concentration (λ = 265.9 nm) in the resulting suspension was analyzed using a flameless atomic absorption spectrometer (AAS), and an AAS platinum standard (Sigma).

### Statistical analysis

Statistical significance was calculated using a two-tailed Fisher's exact test, an ordinary ANOVA, and InStat software (Graphpad, Los Angeles, CA, USA). A two-sided *p*-value of < 0.05 was considered to indicate statistical significance.
